# Pharmacological Evaluation of LiuWei Zhuanggu Granules in Rats

**DOI:** 10.3390/molecules17078001

**Published:** 2012-07-03

**Authors:** Haiming Zhu, Liang Ding, Haijun Xiao, Weifeng Ni, Feng Xue, Zhiming He

**Affiliations:** Department of Orthopaedic Surgery, Shanghai Fengxian Central Hospital, No. 9588, NanFeng Road, Fengxian District, Shanghai 201400, China; Email: haimzhu@126.com (H.Z.); ldingphg@126.com (L.D.); hjxiaojkt@126.com (H.X.); wfnifgh@126.com (W.N.); fxuefgts@126.com (F.X.)

**Keywords:** LWZGG, Ca, Zn, Cu, CT, BGP, uterine

## Abstract

Many commonly consumed foods, herbs and spices contain a complex array of naturally occurring bioactive molecules called phytochemicals, which may confer health benefits. In this study, the impact of LiuWei Zhuanggu Granules (LWZGG) on mineral metabolism in osteopenia development was evaluated. Results showed that serum estrogen, bone gla protein (BGP), and calcitonin (CT) levels, bone Ca, Zn and Cu levels, femur, lumbar vertebrae and trabecular bone density, tibia maximum stress and maximum bending strength were increased, and serum parathyroid hormone (PTH), serum and urine Ca, Zn and Cu levels were decreased in rat bone. It can be concluded that LWZGG is useful to improve bone quality in ovariectomized (OVX) rats.

## 1. Introduction

The incidence of osteoporosis increases dramatically with life expectancy. This disease is characterized by a reduction in bone mass and microarchitectural deterioration of bone tissue, resulting in skeletal fragility and susceptibility to fractures [1,2]. Because hypoestrogenemia after menopause is an important cause of osteoporosis, hormone replacement therapy (HRT) used to be a popular regime for the prevention and treatment of postmenopausal osteoporosis [3,4]. Estrogen deficiency causes increased activation frequency for bone remodeling [5]. The chief consequences are an increase in osteoclasts and resorption lacunae. There is also evidence that the reduced levels of estrogen decrease bone formation, albeit to a lesser extent than the increase in bone resorption [6]. Current treatments include hormonal, pharmacological and mechanical strain interventions. Hormonal and pharmacological interventions are often associated with adverse side effects and target the skeleton as a whole, as opposed to specifically targeting skeletal sites at increased risk for failure. Mechanical strain interventions, however, are noninvasive and have demonstrated promising results. *In vivo* studies have shown low-magnitude, highfrequency vibrations to be anabolic in both human [7] and animal models [8].

Through thousands of years of human experimentation, belief in the safety of “natural” products has contributed to the fairly widespread use of complementary therapies among women to relieve postmenopausal symptoms [9]. Indeed, many of commonly consumed foods, herbs and spices contain a complex array of naturally occurring bioactive molecules called phytochemicals, which may confer health benefits [10,11]. LiuWei Zhuanggu Granules is a Chinese medicine which has been applied to the clinical therapy of bone diseases. LiuWei Zhuanggu Granules contain yak’s bone marrow, Chinese caterpillar fungus, ginseng fruit, *Lycium chinensis* and many useful mineral elements (Ca, P, Fe, Zn). LiuWei Zhuanggu Granules can repair bone damage, increase cells activities, synovial fluid and soft tissue production. In this study, we investigate the effect of LiuWei Zhuanggu Granules on bone quality in OVX rats.

## 2. Results and Discussion

The present study is the first to evaluate the effect of LWZGG on osteoporosis induced by ovariectomy. It is well known that estrogen deficiency is an important risk factor in the pathogenesis of osteoporosis. Ovariectomy in the rat results in an increase in bone turnover rate and significant loss of cancellous bone such as the proximal femur, vertebral bodies and the metaphysis of long bones [12]. The micro architectural alteration in cancellous bone is similar to those observed in postmenopausal women [13,14].

In the present study, as shown in Table 1, there were no statistical differences in uterine wet weight between the NC group and sham group by the end of the experimental period. However, ovariectomy significantly decreased the uterine wet weight in OVX group compared with the sham group (*p* < 0.01). The LWZGG treatments (1 g and 2.5 g/kg b.w.) dose-dependently significantly increased the uterine wet weight in OVX+LWZGG groups compared with the OVX group (*p* < 0.01).

**Table 1:** Uterine wet weight in different groups.

Group	Uterine wet weight (mg)
NC	1048.3 ± 132.5
Sham	1072.1 ± 89.5
OVX	205.3 ± 14.6 ^d^
OVX + LWZGG (1 g/kg b.w.)	369.1 ± 27.7 ^f^
OVX + LWZGG (2.5 g/kg b.w.)	482.5 ± 31.5 ^f^

^d^ *p* < 0.01, compared with sham group; ^f^ *p* < 0.01, compared with OVX group.

Tobias and Compston [15] suggested that estrogen can stimulate osteoblast function and perhaps perform an anabolic action. They suggested that antireabsorptive drugs such as estrogen would be interesting to use in high doses in certain relatively short-term situations, such as the development of osteoporosis in the early postmenopausal period. Serum BGP content is positively correlated with bone BGP content. It can accurately reflect the activity of osteoblasts, and is a marker of osteogenesis [16,17]. Parathyroid hormone (1–34) (PTH) has stimulating effects on osteoblasts and osteoclasts, and may still activate renal α-hydroxylase and promote 1,25-(OH)2D3 synthesis, and as a result, indirectly promote intestinal calcium absorption. PTH, when administered as a daily injection, stimulates bone growth in various species, including osteoporotic women [18,19,20,21,22,23,24]. Excessive PTH secretion may increase bone turnover rate, decomposition and absorption effects. Calcitonin (CT) is an antiresorptive agent, however, its effect on osteoclasts is not continuous when administered in a certain manner, and it is thought that CT therapy maintains physiological bone turnover [25,26,27]. CT may decrease osteoclasts number and activity through its receptor mediated effect on osteoclasts. As a result, CT can modulate activity of osteoblasts and promote bone formation process.

In this study, as shown in Table 2, there were no statistical differences in serum estrogen, PTH and CT levels between the NC group and sham group by the end of the experimental period. Serum BGP level in sham group was significantly (*p* < 0.05) higher than that in the NC group. However, ovariectomy significantly decreased serum estrogen, BGP, CT levels and enhanced the PTH level in OVX group compared with the sham group (*p* < 0.01). The LWZGG treatments (1 g and 2.5 g/kg b.w.) dose-dependently significantly enhanced serum estrogen, BGP, CT levels and decreased the PTH level in OVX+LWZGG groups compared with the OVX group (*p* < 0.01). We suppose that LWZGG might decrease the glucocorticoid antagonism effect on bone metabolism, and prevent steroid-induced osteoporosis effect through increasing serum estrogen, BGP, CT levels, decreasing PTH level, inhibiting the activity of osteoclasts, and improve bone metabolism.

**Table 2:** Serum estrogen, BGP, PTH and CT levels in different groups.

Group	Estrogen (ng/L)	BGP (μg/L)	PTH (ng/L)	CT (μg/L)
NC	4.71 ± 0.29	3.97 ± 0.28	2.08 ± 0.21	521.53 ± 46.27
Sham	4.63 ± 0.37	4.05 ± 0.35 ^a^	1.98 ± 0.16	541.08 ± 38.91
OVX	2.91 ± 0.22 ^d^	1.62 ± 0.73 ^d^	6.89 ± 0.53 ^d^	257.57 ± 32.38 ^d^
OVX+LWZGG (1 g/kg b.w.)	3.78 ± 0.29 ^e^	2.28 ± 0.56 ^f^	4.81 ± 0.43 ^f^	402.48 ± 40.81 ^e^
OVX+LWZGG (2.5 g/kg b.w.)	4.83 ± 0.34 ^f^	3.37 ± 0.31 ^f^	2.94 ± 0.22 ^f^	514.8 ± 44.05 ^f^

^a^ *p* < 0.01, compared with NC group; ^d^ *p* < 0.01, compared with sham group; ^e^ *p* < 0.05, ^f^ *p* < 0.01, compared with OVX group.

As shown in Table 3, there were no statistical differences in serum, bone and urine Ca, Zn and Cu levels between the NC group and sham group by the end of the experimental period. However, ovariectomy significantly decreased the bone Ca, Zn and Cu levels, and increased serum and urine Ca, Zn and Cu levels in the OVX group compared with the sham group (*p* < 0.01). The LWZGG treatments (1 g and 2.5 g/kg b.w.) dose-dependently significantly increased the bone Ca, Zn and Cu levels and decreased serum and urine Ca, Zn and Cu levels in OVX+LWZGG groups compared with the OVX group (*p* < 0.01). This indicated that BSZGG can decrease serum and urine Ca, Zn and Cu loss, and increase bone Ca absorption. Cu and Zn are important components of many enzymes, which play important roles in bone metabolism. Deficiency of Zn may retard growth, and is correlated with decreased bone quality in postmenopausal women [28]. Deficiency of Cu may affect the activity of osteoblasts [29,30,31,32]. Our work showed that LWZGG may enhance bone Cu and Zn content. This is useful for preventing osteoporosis development in OVX rats.

**Table 3:** Serum, bone and urine Ca, Zn and Cu levels in different groups.

Group	Ca			Zn			Cu		
	Serum (mmol/L)	Bone (mg/g)	Urine (mg/g)	Serum (μmol/L)	Bone (μg/g)	Urine (μg/g)	Serum (μmol/L)	Bone (μg/g)	Urine (μg/g)
NC	2.29 ± 0.15	306.71 ± 24.63	1.572 ± 0.131	13.61 ± 1.09	161.29 ± 12.35	2.516 ± 0.21	20.41 ± 1.48	6.04 ± 0.53	0.225 ± 0.016
Sham	2.32 ± 0.18	298.64 ± 20.07	1.603 ± 0.132	12.99 ± 0.97	158.38 ± 11.37	2.603 ± 0.132	21.83 ± 1.08	5.99 ± 0.47	0.231 ± 0.013
OVX	3.06 ± 0.23	236.17 ± 18.59	2.135 ± 0.165	18.82 ± 0.89	113.25 ± 9.54	3.361 ± 0.247	29.77 ± 1.85	4.61 ± 0.41	0.295 ± 0.021
OVX+LWZGG (1 g/kg b.w.)	2.78 ± 0.17	268.14 ± 21.74	1.893 ± 0.111	16.05 ± 1.04	136.19 ± 8.46	3.063 ± 0.224	25.72 ± 2.07	5.45 ± 0.45	0.264 ± 0.019
OVX+LWZGG (2.5 g/kg b.w.)	2.41 ± 0.19	299.21 ± 20.53	1.672 ± 0.132	14.07 ± 0.95	159.04 ± 11.43	2.639 ± 0.198	22.17 ± 1.64	5.85 ± 0.48	0.241 ± 0.016

As shown in Table 4, there were no statistical differences in lumbar vertebrae and trabecular bone density between the NC group and sham group by the end of the experimental period. Femur density in sham group was significantly (*p* < 0.05) lower than that in the NC group. OVX resulted in a significantly decrease in the femur, lumbar vertebrae and trabecular bone density in the OVX group compared with the sham group (*p* < 0.01, Table 4). Compared with the OVX group, 20 weeks of treatments with two doses of LWZGG (1 g and 2.5 g/kg b.w.) significantly (*p* < 0.05; *p* < 0.01) increased the femur, lumbar vertebrae and trabecular bone density in OVX+LWZGG groups dose-dependently (Table 4).

**Table 4:** Femur, lumbar vertebrae and trabecular bone density in different groups.

Group	Femur density (g/cm^2^)	Lumbar vertebrae density (g/cm^2^)	Trabecular bone density (mm^2^ %)
NC	0.3937 ± 0.0276	0.3927 ± 0.0333	0.9143 ± 0.0572
Sham	0.3617 ± 0.0283 ^a^	0.4181 ± 0.0318	0.9188 ± 0.0739
OVX	0.3052 ± 0.0291 ^d^	0.3164 ± 0.0274 ^d^	0.6936 ± 0.0516 ^d^
OVX+LWZGG (1 g/kg b.w.)	0.3241 ± 0.0216 ^e^	0.3719 ± 0.0233 ^f^	0.8159 ± 0.0477 ^f^
OVX+LWZGG (2.5 g/kg b.w.)	0.3379 ± 0.0325 ^f^	0.3946 ± 0.0317 ^f^	0.9052 ± 0.0691 ^f^

^a^ *p* < 0.01, compared with NC group; ^d^ *p* < 0.01, compared with sham group; ^e^ *p* < 0.05; ^f^ *p* < 0.01, compared with OVX group.

The biomechanical competence of the tibia was tested after 20 weeks treatment with LWZGG by using an *ex vivo* three-point bending test. OVX resulted in a significantly decrease in the maximum stress and maximum bending strength in tibia in the OVX group compared with the sham group (*p* < 0.01, Table 5). Compared with the the OVX group, 20 weeks of treatments with two doses of LWZGG (1 g and 2.5 g/kg b.w.) significantly increased the tibia maximum stress and maximum bending strength in OVX+LWZGG groups compared to OVX group (*p* < 0.01, Table 5) dose-dependently.

**Table 5:** Tibia maximum stress and maximum bending strength in different groups.

Group	Tibia maximum stress (MPa)	Tibia maximum bending strength (MPa)
NC	275.6 ± 20.1	321.7 ± 30.5
Sham	271.5 ± 23.6	318.6 ± 28.9
OVX	218.9 ± 18.5 ^d^	268.3 ± 20.4 ^d^
OVX+BSZGG (1 g/kg b.w.)	251.4 ± 20.9 ^f^	293.5 ± 22.2 ^f^
OVX+BSZGG (2.5 g/kg b.w.)	268.3 ± 23.4 ^f^	311.5 ± 28.5 ^f^

^d^ *p* < 0.01, compared with sham group; ^f^ *p* < 0.01, compared with OVX group.

These results indicated that LWZGG may increase bone density, and improve the biomechanical properties of bone. Its mechanism of action might be that LWZGG plays an analogous effect to estrogen, decreasing bone turnover and bone loss in OVX rats.

## 3. Eperimental

### 3.1. Material

LiuWei Zhuanggu Granules (No. Z20025232) were purchased from the Crystal Beads Tibetan Medicine Group (XiNing, China)

### 3.2. Animals and Treatments

Fifty 3-month-old virgin female Sprague-Dawley rats were purchased from the Experimental Animal Center and were acclimated to laboratory conditions for 1 week before the experiment. Animals were housed in a climate controlled room, under a 12 h light/dark photoperiod. All the animals had free access to food and water. The study was approved by the Institutional Animal Care and Use Committee and all of the protocols complied with the Guide for the Care and Use of Laboratory Animals.

The acclimatized rats underwent either bilateral laparotomy (Sham, n = 10) or bilateral ovariectomy (OVX, n = 30). Four weeks after recovering from surgery, the ovariectomized (OVX) rats were randomly divided into three groups: OVX with vehicle (OVX, n = 10); OVX with LWZGG of graded doses (n = 10, 1 g/kg body weight/day) and (n = 10, 2.5 g/kg body weight/day). Another 10 rats served as normal control and were fed with vehicle. Vehicle and LWZGG were all administrated orally through an oral gavage, which started on week 4 after OVX for 20 weeks.

The body weight of the animals was recorded weekly during the experimental period. Urine samples were collected from the rats. After laparotomy using anesthesia with diethyl ether, blood samples were collected via abdominal aorta puncture, and the serum was then prepared by centrifugation of the collected blood (2,000 rpm for 20 min). Urine and serum samples were then stored at −80 °C for biochemical determinations. Uteri were removed from each rat and immediately weighed. Femurs, lumbar vertebrae and trabecular bones were dissected and stored in physiological saline and stored at −20 °C for subsequent measurement.

### 3.3. Biochemical Assay

Serum E2, BGP, PTH and CT levels were determined by a radioimmunoassay method [33,34]. Serum, bone and urine Ca, Zn and Cu levels were determined with an atomic absorption spectrophotometric method [35]. 

### 3.4. BMD Analysis

The bone mineral density (BMD) was measured using a Lunar Prodigy Advance by DXA (GE Healthcare, Madison, WI, USA) using the small laboratory animal scan mode, as reported before [36,37]. The values were calculated automatically by a software package (Encore 2006, GE Healthcare).

### 3.5. Biomechanical Testing Procedure

The mechanical properties of the tibia were measured by a three-point bending test using an MTS instrument (Sintech-1/M, MTS Adamel Lhomargy, Ivry sur Seine, France). Tibias were thawed at room temperature prior to conducting a three-point bending test and continuously moistened with isotonic saline solution. Before performing the three-point bending test, the length of tibia was measured with a digital caliper (Mitutoyo CD-20DC, Mitutoyo Ltd., London, UK) and its midpoint was marked. The tibia was placed on a special holding device with supports located at a distance 18 mm apart. The bending force was applied with the crosshead at a constant speed of 1 mm/min, until maximal load failure and bending stiffness were recorded.

### 3.6. Statistical Analysis

All values from these experiments are expressed as reported as the mean ± standard deviation (S.D.) for each group. Statistical analyses were performed using the statistics package SPSS 13.0 (SPSS, Chicago, IL, USA). Differences among treatment groups were tested by one-way analysis of variance (ANOVA). If significant differences were indicated, differences between the groups were tested by Fisher’s protected least significant difference (PLSD). A *p*-value <0.05 was considered to indicate significant differences.

## 4. Conclusions

LWZGG may enhanced uterine wet weight, serum estrogen, BGP, and CT levels, bone Ca, Zn and Cu levels, increase femur, lumbar vertebrae and trabecular bone density, tibia maximum stress and maximum bending strength, and decrease serum PTH, serum and urine Ca, Zn and Cu levels in OVX rats.
